# Supplementary material for: Conductors’ tempo choices shed light over Beethoven’s metronome
Source: PLoS One. 2020 Dec 16;15(12):e0243616. doi: 10.1371/journal.pone.0243616 (PMC7743971; doi:10.1371/journal.pone.0243616)
Supplement: S1 File — The bmetr R package contains all supporting data and methods. (GZ) [file pone.0243616.s001.gz › bmetr/inst/doc/metr-model.html]

Beethoven’s metronome from photographs


# Beethoven’s metronome from photographs

#### Almudena Martín-Castro, Iñaki Ucar

#### 2020-10-23

This vignette discusses and validates a mathematical model using experimental measurements from a contemporary metronome (see `help(metr.data)`). Then, we fit the metronome model for several metronomes to obtain their nondimensionalized masses, and finally, we characterize Beethoven’s metronome.

```
library(bmetr)
library(errors)
options(errors.warn.coercion=FALSE)
library(dplyr, warn.conflicts=FALSE)
library(ggplot2)
theme_set(ggthemes::theme_tufte(base_family="sans"))
```

**Mechanical metronomes.** From left to right, a contemporary Neewer© NW-707, TB 06, TB 07 (both from Tony Bingham’s *Metronomes and Musical Time*), Maelzel’s patent, and a diagram depicting the main parameters of the model.

# 1 The model

Contemporary mechanical metronomes preserve essentially the same design as Maelzel’s metronome. The angular frequency of oscillation, \(\Omega\), is obtained as a function of three multiplicative terms:

\[
\Omega = f\_\mathrm{ang}^{-1}(\theta)\cdot f\_\mathrm{fric}^{-1}(\epsilon)\cdot \sqrt[]{g\frac{M'R -\frac{\mu'}{2} (l-L) -r}{M'R^2 + \frac{\mu'}{3} ( L^2 + l^2- lL) + r^2}}
\]

where the last term draws from the classical expression for an ideal double pendulum, but includes corrections to account for the effect in the moment of inertia of the non-negligible mass, \(\mu\), of the rod. Other parameters are the gravitational acceleration (\(g\)), the nondimensionalized lower (\(M' = M / m\)) and rod (\(μ' = μ / m\)) masses, the distances of the lower and upper masses to the shaft (\(R\) and \(r\), respectively), and the length of the two ends of the rod from the shaft (\(L\) and \(l\), respectively). The first two terms, \(f\_\mathrm{ang}\) and \(f\_\mathrm{fric}\), are further corrections to account, respectively, for large oscillations (usually ranging from \(θ = 40°\) to \(60°\)) and friction and impulse forces:

\[
f\_\mathrm{ang} (\theta)= 1 + \sum\_{n=1}^\infty\left[ \frac{(2n-1)!!}{(2n)!!}\sin^{2n}\left( \frac{\theta}{2} \right) \right]^2\\
f\_\mathrm{fric} (\epsilon)= 1 + \frac{1}{\pi}\sin^{-1}\left(\frac{\epsilon}{1-\epsilon}\right) - \frac{1}{\pi} \sin^{-1}\left(\frac{\epsilon}{1+\epsilon}\right)
\]

where \(\epsilon = \frac{\tau\_\mathrm{roz}}{\Omega^2 I \theta}\) is a nondimensional parameter that must range from \(0 \leq \epsilon \leq 0.5\), so that the equation has a real solution. It is proportional to the friction torque \(\tau\), and inversely proportional to the angular frequency squared and the moment of inertia.

## 1.1 Measurements

A contemporary metronome was used to validate the model. First, the angular frequency for each metronome mark was measured by means of extracting the tickling period over 15-second audio samples. This package contains a sample file with the audio recorded for 100 bpm.

```
f.raw <- tuneR::readWave(system.file("extdata/100.wav", package="bmetr"))
f.raw
```

```
## 
## Wave Object
##  Number of Samples:      662528
##  Duration (seconds):     15.02
##  Samplingrate (Hertz):   44100
##  Channels (Mono/Stereo): Mono
##  PCM (integer format):   TRUE
##  Bit (8/16/24/32/64):    16
```

These are the steps to measure the frequency:

1. Normalize and square the signal.
2. Remove noise (just zeroing values under 0.5).
3. Find peaks with a sensitivity of 50 ms (see `help(find_peaks)`).
4. Compute the differences (in samples) and transform.

With the sample file:

```
f <- tuneR::normalize(f.raw)^2
f@left[f@left < 0.5] <- 0
peaks <- find_peaks(f@left, f@samp.rate * 0.05)
```

```
tuneR::plot(f.raw)
points(peaks/f@samp.rate, abs(f.raw@left[peaks]), col="red", pch=19)
```

```
period <- diff(peaks) / f@samp.rate
set_errors(60) / set_errors(mean(period), sd(period)/sqrt(length(period)))
```

```
## 96.4(4)
```

The same procedure was repeated with all the files:

```
wav <- Sys.glob(paste0(PATH_WAVS, "/*"))
mark <- as.numeric(sub(".wav", "", basename(wav)))

bpm <- do.call(c, lapply(wav, function(x) {
  f <- tuneR::normalize(tuneR::readWave(x))^2
  f@left[f@left < 0.5] <- 0
  peaks <- find_peaks(f@left, f@samp.rate * 0.05)
  period <- diff(peaks) / f@samp.rate
  set_errors(60) / set_errors(mean(period), sd(period)/sqrt(length(period)))
}))
```

All these measurements are included in the `metr.neewer` data set.

```
head(metr.neewer)
```

```
##   mark      bpm    bpm.se
## 1   40 37.65203 0.6741290
## 2   42 39.50508 0.6750543
## 3   44 41.97662 0.6010792
## 4   46 44.11887 0.5014112
## 5   48 45.99899 0.5032427
## 6   50 47.88837 0.5011762
```

## 1.2 Model accuracy

Then, the metronome was dismantled and all model parameters were measured (dimensions and masses). Our model achieves even better accuracy than the calibration set by the manufacturer (MAE of less than 2 bpm, compared to a MAE of 3 bpm for the metronome scale).

```
# measurements for the masses
# M <- set_errors(30.8, 0.01)
# mu <- set_errors(3.8, 0.01)

# correction for L = R, as for the rest of the metronomes
metr.params[1,]$L <- metr.params[1,]$R
M <- set_errors(31.01, 0.01)
m <- set_errors(7.1, 0.01)
mu <- set_errors(3.59, 0.01)
M. <- M / m
mu. <- mu / m

# gravitational acceleration
g <- set_errors(9.807, 0.04)

# attach rcm, l, R, L, A
metr.params %>%
  filter(model == "Neewer") %>%
  unite_errors() %>%
  attach()

neewer <- metr.marks %>%
  filter(model == "Neewer") %>%
  left_join(metr.neewer, by="mark") %>%
  unite_errors()

comparative <- neewer %>%
  mutate(Model  = metr_model(r+rcm, R, M., l, L, mu., g, A)) %>%
  mutate(Forsen = metr_model(r+rcm, R, M., l, L, 0,   g, 0)) %>%
  gather_errors("series", "value", "Model", "Forsen", "bpm") %>%
  mutate(series = sub("Forsen", "Forsén et al. (2013)", series)) %>%
  mutate(series = sub("bpm", "Experimental", series)) %>%
  mutate(series = reorder(factor(series), -drop_errors(value)))

ggplot(comparative) +
  aes(mark, drop_errors(value), color=series) +
  ggthemes::geom_rangeframe(aes(y=drop_errors(bpm)), data=neewer, color="black") +
  geom_abline(slope=1, color="gray") + geom_point(size=.7) +
  geom_errorbar(aes(ymin=errors_min(value), ymax=errors_max(value)), size=.3) +
  geom_smooth(method="gam", size=.3) +
  labs(x="Metronome mark [bpm]", y="Oscillation frequency [bpm]", color=NULL) +
  theme(legend.position=c(0, 1), legend.justification=c(0, 1),
        axis.title.y=element_text(hjust=0.2), axis.title.x=element_text(hjust=0.5))
```

## 1.3 Effect of corrections

The same contemporary metronome was used to study the effect of each kind of correction. To this end, the true mass of the rod, the true oscillation angle and the maximum friction allowed by the model (\(\epsilon = 0.5\)) were separately compared against the null model (null mass, oscillation angle and friction) along the whole scale range.

```
corrections <- neewer %>%
  mutate(base     = metr_model(r+rcm, R, M., l, L, 0,   g, 0, 0)) %>%
  mutate(Rod      = metr_model(r+rcm, R, M., l, L, mu., g, 0, 0)) %>%
  mutate(Angle    = metr_model(r+rcm, R, M., l, L, 0,   g, A, 0)) %>%
  mutate(Friction = metr_model(r+rcm, R, M., l, L, 0,   g, 0, 0.5)) %>%
  mutate(Rod      = (Rod      - base) / base * 100) %>%
  mutate(Angle    = (Angle    - base) / base * 100) %>%
  mutate(Friction = (Friction - base) / base * 100) %>%
  gather_errors("series", "value", "Rod", "Angle", "Friction") %>%
  mutate(series = factor(series, levels=c("Friction", "Angle", "Rod")))

ggplot(corrections) +
  aes(mark, drop_errors(value), color=series) +
  ggthemes::geom_rangeframe(color="black") +
  geom_point(size=.7) +
  geom_errorbar(aes(ymin=errors_min(value), ymax=errors_max(value)), size=.3) +
  geom_smooth(method="gam", size=.3) +
  labs(x="Metronome mark [bpm]", y="Correction [%]", color="") +
  theme(legend.position=c(0.98, 0.58), legend.justification=c(1, 0))
```

As expected, considering the mass of the rod contributes the most to the model accuracy, and thanks to the escapement wheel, the effect of friction is negligible except for the lowest oscillation frequencies.

# 2 Model transformation and fit

Neglecting the effect of friction (\(f\_\mathrm{fric} \approx 1\)), we express \(\Omega^2\) as a linear combination of polynomial terms of \(r\):

\[
\Omega^2=a\_0 + b\_2 \left(\frac{g}{f\_\mathrm{ang}^2(\theta)} r + \Omega^2 r^2 \right)
\]

where

\[
a\_0= \frac{g}{f\_\mathrm{ang}^2(\theta)} \cdot \frac{M'R-\frac{\mu'}{2}(l-L)}{M'R^2+\frac{\mu'}{3}(L^2+l^2-lL)} \\
b\_2= -\frac{1}{M'R^2+\frac{\mu'}{3}(L^2+l^2-lL)}
\]

## 2.1 Results

This linear model was fitted for two old metronomes from dates similar to Beethoven’s metronome, the patent diagram, and the contemporary metronome as a control (see first figure).

```
metr <- metr.marks
metr[grep("Neewer", metr$model),]$mark <- rep(metr.neewer$bpm, 2)
metr <- metr %>%
  group_by(model) %>%
  slice(-n())

fit <- metr_fit(metr, metr.params) %>%
  unite_errors() %>%
  mutate(model = factor(sub("Neewer", "Control", model)))

pred <- metr_predict(fit) %>%
  cbind(metr[-1]) %>%
  unite_errors() %>%
  mutate(model = factor(sub("Neewer", "Control", model)))

r.squared <- sapply(fit$fit, function(x) summary(x)$adj.r.squared)
labels <- mapply(function(x, y) {
  bquote(.(x)*"," ~ R^2==.(y))
}, levels(pred$model), round(r.squared, 4))

ggplot(pred) +
  aes(drop_errors(r), y, color=reorder(model, drop_errors(r), min)) +
  ggthemes::geom_rangeframe(color="black") +
  geom_ribbon(aes(ymin=lwr, ymax=upr), alpha=.2, size=0) +
  geom_point(size=.7) +
  geom_errorbarh(aes(xmin=errors_min(r), xmax=errors_max(r)), height=2, size=.3) +
  geom_line(aes(y=fit), size=.3) +
  scale_color_discrete(labels=labels) +
  labs(x="r [mm]", y=expression(Omega^2), color=NULL) +
  theme(legend.position=c(1, 1), legend.justification=c(1, 1))
```

Metronome dimensions were measured using Fiji on the basis of the total heights reported in Tony Bingham’s catalogue. The total height is assumed to be 31 cm for the patent according to the patent description and the height of the oldest metronome. The oscillation angle is taken as the maximum inclination, bounded by the box. Parameter R cannot be directly measured for some metronomes (when the box hides the lower mass), so it was estimated taking into account the box size and the patent description. Given that the lower mass hangs approximately from the end of the rod, it is assumed that \(L \approx R\). With these assumptions, we estimated the nondimensional masses, \(M'\) and \(μ'\), for each metronome from the regression coefficients.

```
note <- data.frame(model="Control (true)", M.=M., mu.=mu.)

ggplot(fit) +
  aes(drop_errors(M.), drop_errors(mu.), color=reorder(model, -drop_errors(mu.))) +
  ggthemes::geom_rangeframe(color="black") +
  geom_point(size=.7) +
  geom_point(aes(shape=model), data=note, color="black", size=2) +
  geom_errorbarh(aes(xmin=errors_min(M.), xmax=errors_max(M.)), height=.005, size=.3) +
  geom_errorbar(aes(ymin=errors_min(mu.), ymax=errors_max(mu.)), width=.012, size=.3) +
  scale_shape_manual(name=NULL, values=8) +
  labs(x="Nondimensionalized lower mass", y="Nondimensionalized rod mass", color=NULL) +
  guides(color=guide_legend(order=1), shape=guide_legend(order=2)) +
  theme(legend.position=c(.95, .95), legend.justification=c(1, 1), legend.spacing.y=unit(-2, "mm"))
```

## 2.2 Beethoven’s metronome

Results show that this methodology accurately estimates the masses for the control metronome, and thus, we take the averages of the old metronomes and the patent as a parametrization of Beethoven’s metronome, with the rest of the parameters equal to the measurements for the patent.

```
res <- as.data.frame(fit[3:5, 1:3])
res$model <- as.character(res$model)
res[4,] <- list("Beethoven's metronome (average)", mean(res$M.), mean(res$mu.))
res
```

```
##                             model     M.     mu.
## 1                          Patent 4.1(1) 0.63(2)
## 2                           TB 06 3.9(1) 0.66(3)
## 3                           TB 07 4.1(1) 0.63(3)
## 4 Beethoven's metronome (average) 4.0(1) 0.64(3)
```
