# Supplementary material for: Conductors’ tempo choices shed light over Beethoven’s metronome
Source: PLoS One. 2020 Dec 16;15(12):e0243616. doi: 10.1371/journal.pone.0243616 (PMC7743971; doi:10.1371/journal.pone.0243616)
Supplement: S1 File — The bmetr R package contains all supporting data and methods. (GZ) [file pone.0243616.s001.gz › bmetr/inst/doc/sym-analysis.html]

Beethoven’s symphonies analysis


# Beethoven’s symphonies analysis

#### Almudena Martín-Castro, Iñaki Ucar

#### 2020-10-23

This vignette presents an analysis of the performed tempo for 36 recordings, by 36 different conductors, of Beethoven’s symphonies (see `help(sym.data)`).

```
library(bmetr)
library(errors)
options(errors.warn.bool=FALSE)
library(dplyr, warn.conflicts=FALSE)
library(lme4, warn.conflicts=FALSE)
```

```
## Loading required package: Matrix
```

```
library(ggplot2)
theme_set(ggthemes::theme_tufte(base_family="sans"))
```

# 1 Data set

In this work, we selected 36 recordings of Beethoven’s complete symphonic works as performed by 36 different conductors, and classified them as Historically Informed (HI), under HI influence and Romantic, according to the analysis of L. D. Young (1991) and the performance reviews included with the recordings.

```
knitr::kable(sym.recordings[, c(1, 6, 4, 5, 8)], col.names=c(
  "Conductor", "Dates", "Label", "UPC", "Style"))
```

| Conductor | Dates | Label | UPC | Style |
| --- | --- | --- | --- | --- |
| Abbado, Claudio | 2000-2001 | DG | 028947758648 | HI influence |
| Barenboim, Daniel | 2011 | Decca | 028947835110 | Romantic |
| Bernstein, Leonard | 1977-1979 | DG | 028947492429 | Romantic |
| Böhm, Karl | 1969-1972 | DG | 028947919490 | Romantic |
| Brüggen, Frans | 1984-1992 | Decca | 028947874362 | HI |
| Chailly, Riccardo | 2007-2009 | Decca | 028947834922 | HI influence |
| Cluytens, André | 1957-1960 | Erato | 5099964830353 | Romantic |
| Davis, Colin | 1995 | Philips | 028947568834 | Romantic |
| Ferencsik, Janos | 1969-1976 | Hungaroton | 5991810401321 | Romantic |
| Furtwängler, Wilhelm | 1947-1954 | Andromeda | 3830257490937 | Romantic |
| Gardiner, John Eliot | 1991-1994 | DG | 028943990028 | HI |
| Gielen, Michael | 1997-2000 | Hänssler | 4010276025078 | HI influence |
| Haitink, Bernard | 1985-1987 | Philips | 0028944207323 | Romantic |
| Harnoncourt, Nikolaus | 1990-1991 | Teldec | 0809274976826 | HI influence |
| Hickox, Richard | 1984-1988 | Resonance | 0680125050427 | HI influence |
| Hogwood, Christopher | 1985-1989 | Decca | 028945255125 | HI |
| Hugget, M. and Goodman, R. | 1982-1988 | Nimbus | 0710357514425 | HI |
| Immerseel, Jos Van | 2005-2007 | Zigzag | 3700551732197 | HI |
| Jochum, Eugen | 1967-1969 | Philips | 0028947581475 | Romantic |
| Karajan, Herbert von | 1951-1955 | Warner Classics | 5099951586324 | Romantic |
| Kegel, Herbert | 1982-1984 | Capriccio | 4006408500001 | Romantic |
| Klemperer, Otto | 1960 | Music & Arts | 0017685125225 | Romantic |
| Leinsdorf, Erich | 1961-1969 | RCA | 0886919168228 | Romantic |
| Masur, Kurt | 1972- 1975 | Philips | 0028947527220 | Romantic |
| Mengelberg, Willem | 1940 | Archipel Records | 4035122401929 | Romantic |
| Muti, Riccardo | 1985-1988 | Warner Classics | 5099909794627 | Romantic |
| Norrington, Roger | 1987-1990 | Erato | 5099908342324 | HI |
| Pletnev, Mikhail | 2007 | DG | 0028947764090 | Romantic |
| Polizzi, Antonino | 1986-1994 | Polymnie | 3576079901205 | Romantic |
| Rattle, Simon | 2002 | EMI | 5099991562425 | HI influence |
| Solti, Georg | 1986-1989 | Decca | 0028943040020 | Romantic |
| Szell, George | 1956-1964 | Sony | 0888837371520 | Romantic |
| Toscanini, Arturo | 1949-1952 | RCA | 0828765570220 | Romantic |
| Tremblay, Jean-Philippe | 2009 | Analekta | 0774204997526 | HI influence |
| Walter, Bruno | 1958-1959 | Sony | 5099750231227 | Romantic |
| Wand, Günter | 1985-1988 | RCA | 0743218910920 | Romantic |

The 9th Symphony is exceptional for various reasons. First, it was metronomized seven years later than the others using a different device, of which the date of purchase is not known. Second, its complexity, especially regarding the fourth movement, makes tempo extraction too unstable and unreliable. Finally, some authors have questioned the validity of the documental sources where these tempi were first published, only months before the composer’s death, due to multiple copy mistakes. For all these reasons, we decided to exclude it from the analysis. The rest of the data set comprises 1188 audio files (one symphonic movement per track), more than 169 hours of music.

# 2 Tempo extraction

Audio files were sampled using a sliding window. Its duration was defined as a fraction of the track, so that the average width was 30 seconds, with a 90% overlap. In this way, each symphonic movement is divided in the same number of samples, regardless of the interpreter or the duration of the track. Every sample was then analyzed using a state-of-the-art tempo extraction algorithm by G. Percival et al. (2013, 2014) that bases pulse detection on self-similarity relations within the rhythm of a musical recording, and is implemented as part of the open-source framework Marsyas.

This package contains all the auxiliary scripts used to extract tempo from the original recordings following this procedure:

```
list.files(system.file("scripts", package="bmetr"))
```

```
## [1] "config.sh"           "launcher.sh"         "sym_sample_end.sh"  
## [4] "sym_sample_ratio.sh" "sym_sample_start.sh" "sym_tutti.sh"       
## [7] "sym_window.sh"       "tempo_sample.sh"
```

The result is the following data set:

```
head(sym.window)
```

```
##   symphony movement       conductor n     start  duration tempo
## 1        1        1 Abbado, Claudio 0  0.000000 544.41796    96
## 2        1        1 Abbado, Claudio 1  0.000000  30.24544   139
## 3        1        1 Abbado, Claudio 2  3.024544  30.24544   172
## 4        1        1 Abbado, Claudio 3  6.049088  30.24544   113
## 5        1        1 Abbado, Claudio 4  9.073633  30.24544   153
## 6        1        1 Abbado, Claudio 5 12.098177  30.24544   122
```

## 2.1 First steps

Sections containing a change of tempo or meter were identified and located on the score and the resulting samples. Different sections and movements were classified according to their meter (simple, duple or triple meter, with binary or ternary subdivision). This classification is important in order to identify the most probable tempo harmonics detected by the tempo extraction algorithm for each sample.

**Most common tempo harmonics for each kind of meter**. The tempo extraction algorithm relies on periodic patterns and rhythmic self-similarities. This explains why many of its estimated tempi are actually multiples or submultiples of the real tempo of the sample. In this work, we have called these kinds of mistaken tempi “harmonics” due to the similarity with the homonym physical phenomenon. Their most common values depend on the metric structure of the music and are displayed here. More rarely, we also detected: (i) harmonics 2 y 3/4 in compound meters; (ii) harmonics 2 y 3/4 in meters with binary subdivision due to the occasional use of triplets; (iii) harmonic 2/3, in triple meters with binary subdivision.

First, we annotate different section numbers based on the scores.

```
dt.window <- sym.window %>%
  # filter out estimation for the complete movement
  filter(n != 0) %>%
  # annotate sections
  mutate(section = case_when(
    (symphony==1 & movement==1 & n<22) |
      (symphony==1 & movement==4 & n<6) |
      (symphony==2 & movement==1 & n<51) |
      (symphony==3 & movement==4 & n>130 & n<206) |
      (symphony==4 & movement==1 & n<55) | 
      (symphony==4 & movement==3 & ((n>37 & n<57) | (n>85 & n<105))) | 
      (symphony==5 & movement==2 & n>158 & n<179) | 
      (symphony==5 & movement==4 & n>90 & n<110) | 
      (symphony==6 & movement==3 & ((n>29 & n<46) | (n>77 & n<94))) | 
      (symphony==7 & movement==1 & n<80) | 
      (symphony==7 & movement==3 & ((n>43 & n<78) | (n>112 & n<145) | n>174)) |
      (symphony==9 & movement==2 & ((n>110 & n<165) | (n>236))) | 
      (symphony==9 & movement==3 & ((n>43 & n<70) | (n>113 & n<130)))
    ~ 2,
    (symphony==5 & movement==4 & n>170) | 
      (symphony==3 & movement==4 & n>205)
    ~ 3,
    TRUE ~ 1
  ))
```

We filter out erroneous data and sections that are too short to give meaningful results.

```
dt.window <- dt.window %>%
  filter(
    # invalid tempo data
    tempo != 0,
    # 4th mvt. for symphonies 3 and 5, too fast and short
    !(section==3),
    # slow starts
    !(symphony==1 & section==2),
    !(symphony==2 & movement==1 & section==2 & n<15),
    # 9th symphony, due to reasons above
    !(symphony==9),
    # very short section, irrelevant
    !(symphony==5 & movement==2 & section==2),
    # issues in the audio file
    !(symphony==6 & movement==4 & conductor=="Cluytens, André" & n<11)
  )
```

From the histograms of tempi, we calculate the most prominent tempo (peak of the histogram) per symphony, movement and section (`p.s`), the average duration (`avgdur`) and the most prominent tempo per symphony, movement, section and conductor (`p.sc`).

```
dt.window <- dt.window %>%
  # p.s = peak per section
  group_by(symphony, movement, section) %>%
  mutate(p.s = tmp_prevalent(tempo)) %>%
  # exceptions checked by manual inspection
  mutate(p.s = ifelse(symphony==1 & movement==3, tmp_prevalent(tempo[1:25]), p.s)) %>%
  mutate(p.s = ifelse(symphony==3 & movement==3, 2*tmp_prevalent(tempo[1:47]), p.s)) %>%
  mutate(p.s = ifelse(symphony==3 & movement==2, tmp_prevalent(tempo[140:175]), p.s)) %>%
  mutate(avgdur = mean(duration)) %>%
  
  # p.sc = peak per section and conductor
  group_by(symphony, movement, section, conductor) %>%
  mutate(p.sc = tmp_prevalent(tempo)) %>%
  # exceptions checked by manual inspection
  mutate(p.sc = ifelse(symphony==1 & movement==3, tmp_prevalent(tempo[1:25]), p.sc)) %>%
  mutate(p.sc = ifelse(symphony==3 & movement==3, tmp_prevalent(tempo[1:47]), p.sc)) %>% 
  mutate(p.sc = ifelse(symphony==3 & movement==2, tmp_prevalent(tempo[140:175]), p.sc)) %>%
  
  ungroup()
```

Corrections to `p.s`:

```
dt.window <- dt.window %>%
  mutate(p.s = case_when(
    (symphony==1 & movement==1 & section==2) |
      (symphony==1 & movement==4 & section==2) |
      (symphony==3 & (movement==2 | movement==4) & section==1) | 
      (symphony==4 & movement==4) | 
      (symphony==6 & movement==1) |
      (symphony==7 & movement==1 & section==2) |
      (symphony==7 & movement==2) | 
      (symphony==8 & movement==4)
    ~ p.s / 2,
    (symphony==2 & movement==4) | 
      (symphony==6 & movement==3 & section==2) |
      (symphony==7 & movement==3 & section==1)
    ~ p.s * 2,
    (symphony==6 & movement==2) | 
      (symphony==3 & movement==1) |
      (symphony==6 & movement==5)
    ~ p.s / 3,
    symphony==6 & movement==3 & section==1
    ~ p.s / (3/2),
    TRUE ~ p.s
  ))
```

We calculate `p.sce` as an enhanced version of `p.sc` by various methods:

```
dt.window <- dt.window %>%
  # add annotations and define p.sce
  left_join(sym.marks, by=c("symphony", "movement", "section")) %>%
  mutate(p.sce = p.sc) %>%
  
  # flag whether there's any repetition in each movement
  group_by(symphony, movement) %>%
  mutate(mov.rep = sum(bar.rep) > 0) %>%
  ungroup() %>%
  
  # p.sd = p.s corrected with respect to the avgdur
  # it depends on whether there are repetitions in that movement
  mutate(p.sd = if_else(
    !mov.rep,
    p.s*avgdur/duration,
    p.s*(1 + 0.5*(avgdur/duration-1)*(cos(pi*(avgdur/duration-1)))^2)
  )) %>%
  
  # p.sce must be close to p.sd
  group_by(symphony, movement, conductor, section) %>%
  mutate(p.sce = tmp_rectify_tsig(p.sce, p.sd, tsig, 0.18, 0.187)) %>%
  
  # additional corrections to p.sce
  mutate(p.sce = case_when(
    tsig==3 & p.s>90 | 
      (symphony==2 & movement==1 & section==2 & conductor=="Hogwood, Christopher")
      ~ tmp_rectify(p.sce, p.sd, 3/4, 0.13, 0.114),
    tsig==3 & p.s<90 ~ tmp_rectify(p.sce, p.sd, 2, 0.12, 0.185),
    TRUE ~ p.sce
  )) %>%
  mutate(p.sce = if_else(mov.rep, tmp_rectify_tsig(p.sce, p.s, tsig, 0.18, 0.22), p.sce)) %>%
  mutate(p.sce = case_when(
    symphony==2 & movement==1 & section==2 & conductor=="Rattle, Simon" ~ p.sc*0.85,
    (symphony==3 & movement==4 & section==2) |
      (symphony==2 & movement==1 & section==2) ~ tmp_rectify(p.sce, p.sd, 3/2, 0.144, 0.1),
    symphony==8 & movement==3 & section==1 ~ tmp_rectify(p.sce, p.sd, 2/3, 0.1, 0.1),
    # exceptions checked by manual inspection
    symphony==1 & movement==1 & section==2 & p.sce>100 ~ p.sce / 2,
    symphony==3 & movement==2 & section==1 & conductor=="Furtwängler, Wilhelm" ~ p.sc / 2,
    symphony==7 & movement==3 & section==1 & conductor=="Klemperer, Otto" ~ p.sc,
    symphony==6 & movement==1 & section==1 & conductor=="Pletnev, Mikhail" ~ p.sc / 2,
    TRUE ~ p.sce
  )) %>%
  
  # modify some p.sce due to general tempo variations
  mutate(p.sce = case_when(
    (symphony==1 & movement==3 & n>25 & n<51 & !grepl("Brüggen|Abbado|Hickox", conductor)) | 
      (symphony==3 & movement==2 & (n<70 | (n>129 & n<140) | n>225)) |  
      (symphony==3 & movement==2 & n>190 & conductor=="Tremblay, Jean-Philippe") |
      (symphony==3 & movement==3 & n>40 & n<78 & conductor=="Mengelberg, Willem") |
      (symphony==3 & movement==3 & n>49 & n<78) |  
      (symphony==6 & movement==4 & n>55) 
    ~  p.sce*0.85,
    (symphony==2 & movement==3 & n>30 & n<48 & conductor=="Pletnev, Mikhail")  ~ p.sce*0.75,
    (symphony==1 & movement==3 & n>40 & n<51 & conductor=="Hickox, Richard") |
      (symphony==2 & movement==3 & n>27 & n<46 & conductor=="Harnoncourt, Nikolaus") |
      (symphony==3 & movement==2 & (n<130 | n>170) & !grepl("Leinsdorf|Gardiner", conductor)) |
      (symphony==6 & movement==2 & n<150) |
      (symphony==8 & movement==3 & n>40 & n<71 & conductor!="Furtwängler, Wilhelm")
    ~ p.sce*0.9,
    (symphony==7 & movement==2 & n>85 & n<135)  ~ p.sce*1.15,
    (symphony==5 & movement==3 & n<34) ~ p.sce*(1 + 0.1*(1 - n/100)),
    TRUE ~ p.sce
  )) %>%
  
  ungroup()
```

## 2.2 Data cleaning

In the following, `p.sce` is used as a reference to find and rectify harmonics in the histogram within a certain tolerance. Tolerances are defined case per case to avoid harmonics getting mixed.

```
# we will take a representative example for 3 conductors
example.filter <- quo(
  symphony==3 & movement==1 & grepl("Wand|Chailly|Immerseel", conductor))
# raw tempo data, before any filtering
example.window <- list(filter(dt.window, !!example.filter))

dt.window <- dt.window %>%
  group_by(symphony, movement, conductor, section) %>%
  
  # harmonics 3 and 1/3 (extreme cases)
  mutate(tempo = tmp_rectify_tsig(tempo, p.sce, tsig, 0.14, 0.17))  %>%
  mutate(tempo = tmp_rectify(tempo, p.sce, 3, 0.19, 0.5)) %>% 
  mutate(tempo = tmp_rectify(tempo, p.sce, 1/3, 0.5, 0.15)) %>%

  # typical harmonics by time signature
  mutate(tempo = case_when(
     tsig!=3  ~ tmp_rectify(tempo, p.sce, 2, 0.18, 0.5),
     tsig==3 & p.s<90 ~ tmp_rectify(tempo, p.sce, 2, 0.133, 0.15), 
     tsig==3 & p.s>90 ~ tmp_rectify(tempo, p.sce, 3/4, 0.13, 0.12),
     TRUE ~ tempo
  )) %>% 
  
  # harmonic 3/2
  mutate(tempo = case_when(
    (symphony==1 & movement==2 & ((n>36 & n<80) | n>110)) |
      (symphony==2 & movement==1) |                   # tsig 2
      (symphony==2 & movement==2 & section==1) |      # tsig 0.3
      (symphony==3 & movement==2 & ((n>66 & n<130) | (n>175 & n<230))) | # tsig 2
      (symphony==3 & movement==4 & section!=3) |      # tsig 2
      (symphony==4 & movement==2) |                   # tsig 0.3
      (symphony==5 & movement==2)  |                  # tsig 0.3
      (symphony==7 & movement==1 & section==2) |      # tsig 2
      (symphony==7 & movement==2 & ((n>44 & n <84) | n>133)) |
      (symphony==8 & movement==2 & n>65) |            # tsig 2
      (symphony==8 & movement==3 & n>38 & n<81) |     # tsig 0.3
      (symphony==8 & movement==4)                     # tsig 2
    ~ tmp_rectify(tempo, p.sce, c(3/4, 3/2), 0.12, 0.14),
    TRUE ~ tempo
  )) %>% 
  
  # harmonic 2/3
  mutate(tempo = case_when(
    tsig==0.3 | 
      (symphony==2 & movement==1) |                 # tsig 2
      (symphony==2 & movement==4) |
      (symphony==3 & movement==2) |                 # tsig 2
      (symphony==5 & movement==1)                   # tsig 2
    ~ tmp_rectify(tempo, p.sce, 2/3, 0.12, 0.115),
    TRUE ~ tempo
  )) %>% 
  
  # harmonics 2, 1/2, 4/3
  mutate(tempo = case_when(
    (symphony==2 & movement==4) |                # tsig 2
      (symphony==5 & movement==4 & section==3)   # tsig 2
    ~ tmp_rectify(tempo, p.sce, 1/2, 0.5, 0.21),
    (symphony==2 & movement==1 & section=="1") |
    (symphony==4 & movement==1 & section=="1") | 
      (symphony==7 & movement==1 & section==2 & conductor !="Pletnev, Mikhail")
    ~ tmp_rectify(tempo, p.sce, 4/3, 0.1, 0.15), 
    (symphony==1 & movement==1) |
      (symphony==1 & movement==4 & n>100) |
      (symphony==2 & movement==1 & n<15) |
      (symphony==3 & movement==1) |
      (symphony==4 & movement==3 & section==2) |
      (symphony==6 & movement==2) |
      (symphony==6 & movement==5) |
      (symphony==7 & movement==1) |
      (symphony==7 & movement==3 & section==2) |
      (symphony==8 & movement==1) 
    ~ tmp_rectify(tempo, p.sce, 2, 0.2, 0.5),
    TRUE ~ tempo
  )) %>%
  
  ungroup()

# tempo data after harmonic correction
example.window <- c(example.window, list(filter(dt.window, !!example.filter)))
```

Then, tempo values are corrected using a continuity criterion. In a typical recording, tempo can vary a lot, so the harmonics correction based on the histogram peak might sometimes fail. In those cases we can take advantage of the fact that tempo usually varies smoothly: each data point is compared with the previous 3 samples in search for the same harmonic relationships as in the previous step, and corrected appropriately if found. Finally, outliers, defined as data points that differ more than 2 standard deviations from the corrected peak, are removed and replaced by interpolated values.

```
dt.window <- dt.window %>%
  group_by(symphony, movement, conductor, section) %>%
  
  # continuity based on time signature
  mutate(tempo = tmp_smooth(tempo, p.sce, tsig, 0.12, 0.15)) %>%

  # special cases harmonic 3
  mutate(tempo = if_else(
    !(symphony==7 & movement==4 & conductor=="Mengelberg, Willem" & n>53) &
      !(symphony==5 & movement==2 & conductor=="Jochum, Eugen" & n>109 & n <121) &
      !(symphony==3 & movement==4 & conductor=="Wand, Günter" & n>155 & n <165),
    tmp_smooth(tempo, p.sce, 3, 0.12, 0.12), tempo)) %>%
  
  # special cases harmonic 2/3
  mutate(tempo = if_else(
    (symphony==8 & movement==3),
    tmp_smooth(tempo, 0.95*p.sce, 0, 0.12, 0.12, 2/3), tempo)) %>% 
  
  # special cases harmonic 3/4
  mutate(tempo = if_else(
    tsig==3 | (symphony==1 & movement==2 & ((n>5 & n<87) | n>110)),
    tmp_smooth(tempo, p.sce, 0, 0.12, 0.12, 3/4), tempo)) %>%
  
  # remove outliers and interpolate
  mutate(sd = sd(tempo)) %>%
  mutate(tempo = if_else(tempo > p.sce + 2*sd, NA_real_, tempo)) %>%
  mutate(tempo = if_else(tempo < p.sce - 2*sd, NA_real_, tempo)) %>%
  mutate(tempo = zoo::na.approx(tempo, na.rm=FALSE)) %>%
  ungroup() %>% na.omit() %>%
  
  # recalculation of p.s and p.sce
  group_by(symphony, movement, section) %>%
  mutate(p.s = tmp_prevalent(tempo, 50)) %>%
  group_by(symphony, movement, conductor, section) %>%
  mutate(p.sce = tmp_prevalent(tempo, 50)) %>%
  ungroup()

# tempo data after continuity correction
example.window <- c(example.window, list(filter(dt.window, !!example.filter)))
```

These are the results of this data cleaning process:

```
example.window <- bind_rows(example.window, .id="stage")
notes <- data.frame(
  mark = rep(unique(example.window$mark), 5) * c(rep(1, 3), 3/2, 3),
  label = paste0("Beethoven's mark", c(rep("", 3), " x3/2", " x3")),
  vjust = c(rep(-.6, 4), 1.6),
  stage = c(3, 2, 1, 1, 1))
breaks <- c(40, 50, 60, 80, 120, 160)
expand <- c(39, 70)
balpha <- 0.5

g <- ggplot(example.window) +
  aes(n, tempo, color=conductor) +
  ggthemes::geom_rangeframe(color="black") +
  geom_hline(aes(yintercept=mark), notes, alpha=balpha, linetype=2) +
  facet_grid("stage", scales="free_y", space="free_y") +
  geom_line(alpha=1) + expand_limits(y=expand) +
  scale_y_continuous(breaks=breaks) +
  labs(y="Tempo [bpm]", x="Sample") + guides(color=FALSE) +
  theme(strip.text=element_blank())

h <- ggplot(example.window) + theme_void() +
  aes(tempo, after_stat(density)) +
  coord_flip() + expand_limits(x=expand) +
  facet_grid("stage", scales="free_y", space="free_y") +
  stat_bin(aes(fill=conductor), position="identity",
           alpha=.7, binwidth=1, boundary=40) +
  geom_vline(aes(xintercept=mark), notes, alpha=balpha, linetype=2) +
  geom_text(aes(mark, 0.32, label=label, vjust=vjust), notes,
            hjust=1, alpha=balpha, size=3) +
  scale_x_continuous(breaks=breaks) + labs(fill="Conductor") +
  guides(color=FALSE, fill=guide_legend(title=, override.aes=list(alpha=1))) +
  theme(legend.position=c(1, .77), legend.justification=c(1, 1),
        legend.key.size=unit(5, "pt"), legend.title=element_text(size=10),
        legend.text=element_text(size=8), strip.text=element_blank())

patchwork::wrap_plots(g, h, nrow=1, widths=c(5, 1.5))
```

The top panel shows a representative example of raw data from the tempo extraction algorithm for 3 different conductors performing the 1st movement of the 3rd Symphony. Although the time series seem noisy on first sight, the histogram in the right panel shows a clear pattern: due to the rhythmic complexity of Beethoven’s music, the algorithm not only detects the true tempo (components right below Beethoven’s mark), but also multiples (or harmonics) of this frequency (in this example, x3/2 and x3). Using Beethoven’s mark as a reference for the “true tempo”, harmonics in the raw data are found and rectified (central panel). The final smoothing ensures consistency in terms of continuity throughout contiguous samples (bottom panel).

## 2.3 Validation

A complementary methodology was developed to assess the validity of this collection of tempo measurements. We sampled 30 seconds from the last minute of every movement, thus compiling a set of finales, where tempo is arguably more stable (see `help(sym.sample`). Tempo was also extracted using Marsyas on first pass, but then carefully curated by hand. The main data set is validated by comparing the median tempo for each conductor and mark with the median tempo as obtained from this data set of finales.

```
dt.sample <- sym.sample %>%
  left_join(sym.marks, by=c("symphony", "movement", "section"))

dt.val.conductor <- dt.window %>%
  group_by(conductor) %>%
  summarise(window = median(tempo - mark, na.rm=TRUE)) %>%
  left_join(
    dt.sample %>%
      group_by(conductor) %>%
      summarise(sample = median(tempo - mark, na.rm=TRUE))) %>%
  left_join(sym.recordings[,c(1, 8)], by="conductor") %>%
  mutate(ptype = droplevels(ptype, "Romantic"))
```

```
## Joining, by = "conductor"
```

```
ggplot(dt.val.conductor) +
  aes(window, sample) + ggthemes::geom_rangeframe() +
  geom_abline(color="lightgray") +
  geom_point(aes(color=ptype)) + geom_smooth(method=lm, formula=y~x) +
  ggpmisc::stat_poly_eq(formula=y~x, parse=TRUE) +
  scale_color_discrete(breaks=levels(factor(dt.val.conductor$ptype))) +
  labs(x="Main data set", y="Validation data set", color="Performance",
       subtitle="Median tempo difference by conductor [bpm]") +
  theme(legend.position=c(1, 0), legend.justification=c(1, 0))
```

```
dt.val.mark <- dt.window %>%
  group_by(mark) %>%
  summarise(window = median(tempo, na.rm=TRUE)) %>%
  left_join(
    dt.sample %>%
      group_by(mark) %>%
      summarise(sample = median(tempo, na.rm=TRUE)))
```

```
## Joining, by = "mark"
```

```
ggplot(dt.val.mark) +
  aes(window, sample) + ggthemes::geom_rangeframe() +
  geom_abline(color="lightgray") +
  geom_point() + geom_smooth(method=lm, formula=y~x) +
  ggpmisc::stat_poly_eq(formula=y~x, parse=TRUE) +
  labs(x="Main data set", y="Validation data set", color="Performance",
       subtitle="Median tempo by mark [bpm]") +
  theme(legend.position=c(0, 1), legend.justification=c(0, 1))
```

# 3 Results

## 3.1 By conductor

After this process, we obtained a very accurate description of the performed tempi of Beethoven’s symphonies, which supports previous qualitative analyses. Overall, performed tempi are always slower than Beethoven’s marks on average, but the influence of the HI movement is evident: attending to the median of their distribution, 12 out of the 15 fastest interpretations correspond to HI or HI-influenced performers.

```
dt.window <- dt.window %>%
  left_join(sym.recordings[,c(1, 8)], by="conductor") %>%
  mutate(conductor = reorder(conductor, tempo - mark, median, na.rm=TRUE))

dt.window %>%
  mutate(ptype = droplevels(ptype, "Romantic")) %>%
  ggplot() + aes(tempo - mark, conductor) +
  ggridges::geom_density_ridges(
    aes(fill=ptype), color="lightgray", size=0.3,
    quantile_lines=TRUE, quantiles=2, vline_color="white", vline_size=1) +
  geom_vline(xintercept=0, color="black") +
  scale_fill_discrete(breaks=levels(factor(dt.window$ptype))) +
  scale_y_discrete(position="right") + xlim(-40, 20) +
  labs(y=NULL, x="Tempo difference [bpm]", fill="Performance") +
  theme(legend.position=c(0.97, 0.03), legend.justification=c(1, 0), 
        legend.background=element_rect(color="lightgray"), 
        axis.ticks.y=element_blank(), 
        panel.grid.major.y=element_line(color="lightgray", size=0.3))
```

Karl Böhm, at the bottom of the list, is well known among critics as one of the slowest performers of Beethoven, sometimes even characterized as “stiff” (see Carr). On the other end, Riccardo Chailly is the conductor who comes closer to the composer’s indications as he reportedly intended. But even he falls slightly behind Beethoven’s marks. Some critics have even praised this apparent failure. According to Richard Osborne, “happily Chailly is too good a musician to put into practice his reported assertion that he performs everything at ‘precisely Beethoven’s metronome mark’”. Mikhail Pletnev constitutes a remarkable exception, being at times faster than HI performers but also slower than any other Romantic conductor. Although he generally rejects HI criteria, critics consider him an artist of contrasts, unorthodox and unpredictable. As Osborne put it, “For every movement conducted by Dr Jekyll there is another just around the corner awaiting its fate at the hands of Mr Hyde.”

## 3.2 By mark

Performed tempo is modelled as a function of the metronome marks by means of a mixed-effects linear model, using the intercept as a random effect for each conducting style.

```
dt.medians <- dt.window %>%
  group_by(ptype, mark) %>%
  summarise(tempo = median(tempo, na.rm=TRUE))
# all groups agree on this mark, as we'll see
dt.exception <- filter(dt.medians, mark == 72)
dt.medians <- filter(dt.medians, mark != 72)

fit <- lmer(tempo ~ mark + (1 | ptype), dt.medians)
summary(fit)
```

```
## Linear mixed model fit by REML ['lmerMod']
## Formula: tempo ~ mark + (1 | ptype)
##    Data: dt.medians
## 
## REML criterion at convergence: 335.6
## 
## Scaled residuals: 
##      Min       1Q   Median       3Q      Max 
## -2.81362 -0.63705 -0.08981  0.64414  1.91487 
## 
## Random effects:
##  Groups   Name        Variance Std.Dev.
##  ptype    (Intercept) 11.92    3.452   
##  Residual             18.25    4.272   
## Number of obs: 57, groups:  ptype, 3
## 
## Fixed effects:
##             Estimate Std. Error t value
## (Intercept) -8.88736    2.95600  -3.007
## mark         0.98857    0.02188  45.181
## 
## Correlation of Fixed Effects:
##      (Intr)
## mark -0.713
```

```
re <- merTools::REsim(fit)
names(re)[6] <- "mean.se"
re <- unite_errors(re)
re$mean <- fixef(fit)[1] + re$mean
re[, 1:4]
```

```
##   groupFctr      groupID        term   mean
## 1     ptype           HI (Intercept)  -6(2)
## 2     ptype HI influence (Intercept)  -8(2)
## 3     ptype     Romantic (Intercept) -12(2)
```

```
dt.medians <- cbind(dt.medians, merTools::predictInterval(fit, level=0.95))

r2 <- performance::r2(fit)
r2 <- paste0(
  "atop(\"Conditional \"~italic(R)^2~`=`~", round(r2[[1]], 3),
  ",~~~~\"Marginal\"~italic(R)^2~`=`~", round(r2[[2]], 3), ")")
perf <- data.frame(mark=150, tempo=40, ptype="Romantic", label=r2)

ggplot(dt.medians) +
  aes(mark, tempo) + facet_grid(.~ptype) +
  ggthemes::geom_rangeframe(color="black") +
  geom_abline(color="lightgray") +
  geom_violin(aes(group=mark), dt.window, scale="width", color="lightgray") +
  geom_point() + geom_point(data=dt.exception, shape=1) +
  geom_ribbon(aes(ymin=lwr, ymax=upr), alpha=.2) +
  geom_line(aes(y=fit), color="blue") +
  geom_text(aes(label=label), perf, hjust=1, parse=TRUE) +
  labs(x="Metronome mark [bpm]", y="Performed tempo [bpm]") +
  theme(legend.position=c(0, 1), legend.justification=c(0, 1))
```

This model reveals a common trend shared by all groups: a 1:1 relationship with the marks (95% CI [0.95, 1.03]), and a significative random effect (LRT = 15.29, p < .001), which suggests that performers slow down Beethoven’s marks, on average, by a fixed amount that is different for each group of conductors.

## 3.3 Metronome distortions

Hereafter, we consider the average discrepancy measured by this model for Romantic conductors as a proxy for Beethoven’s intended tempo. Thus, we are interested in comparing these results with possible distortions that decrease the metronome’s frequency by a comparable amount throughout all its range, without remarkable defects or anomalous behaviors that could warn Beethoven about a flaw in the device.

```
dt.window.r <- filter(dt.window, ptype=="Romantic")
dt.medians.r <- filter(dt.medians, ptype=="Romantic")
dt.exception.r <- filter(dt.exception, ptype=="Romantic")

# Beethoven's metronome parameters
M. <- set_errors(4.008046, 0.1068220)
mu. <- set_errors(0.6387369, 0.02924174)
metr.params %>%
  filter(model == "Patent") %>%
  unite_errors() %>%
  attach()
```

```
## The following objects are masked from . (pos = 5):
## 
##     A, L, R, l, model, rcm
```

```
p <- ggplot(dt.medians.r) +
  aes(mark, tempo) +
  ggthemes::geom_rangeframe(aes(y=fit), color="black") +
  geom_abline(color="lightgray") +
  geom_line(aes(y=fit), color="red") +
  labs(x="Metronome mark [bpm]", y="Performed tempo [bpm]") +
  theme(legend.position=c(.02, 1), legend.justification=c(0, 1))
```

We analyzed the possible variation of the lower mass M and its distance to the shaft R resulting from some possible blow that could have broken or loosen it up, as proposed by Forsén et al. (2013). However, these are similar distortions that mostly affect the slower frequencies.

```
p + scale_color_gradient(breaks=c(0, -4, -8), name="Variation of R [mm]") +
  stat_function(fun=metr_model_bias, args=list(
    R=c(R, R-2), M.=M., l=l, mu.=mu., A=A), aes(color=-2)) +
  stat_function(fun=metr_model_bias, args=list(
    R=c(R, R-5), M.=M., l=l, mu.=mu., A=A), aes(color=-5)) +
  stat_function(fun=metr_model_bias, args=list(
    R=c(R, R-8), M.=M., l=l, mu.=mu., A=A), aes(color=-8))
```

```
p + scale_color_gradient(breaks=c(0, -10, -15), name="Variation of M [%]") +
  stat_function(fun=metr_model_bias, args=list(
    R=R, M.=c(M., M.-0.05*M.), l=l, mu.=mu., A=A), aes(color=-5)) +
  stat_function(fun=metr_model_bias, args=list(
    R=R, M.=c(M., M.-0.10*M.), l=l, mu.=mu., A=A), aes(color=-10)) +
  stat_function(fun=metr_model_bias, args=list(
    R=R, M.=c(M., M.-0.15*M.), l=l, mu.=mu., A=A), aes(color=-15))
```

We also considered different inclinations of the metronome, maybe held in an unstable position on the piano while rehearsing. This affects the gravitational acceleration experimented by the pendulum, but would have caused the quicker frequencies to decelerate mostly and, more importantly, would only be noticeable for extremely sharp inclinations.

```
p + scale_color_gradient(breaks=c(0, 20, 40), trans="reverse", name="Variation of inclination [°]") +
  stat_function(fun=metr_model_bias, args=list(
    R=R, M.=M., l=l, mu.=mu., A=A, g=9.807*c(1, cos(10*pi/180))), aes(color=10)) +
  stat_function(fun=metr_model_bias, args=list(
    R=R, M.=M., l=l, mu.=mu., A=A, g=9.807*c(1, cos(25*pi/180))), aes(color=25)) +
  stat_function(fun=metr_model_bias, args=list(
    R=R, M.=M., l=l, mu.=mu., A=A, g=9.807*c(1, cos(40*pi/180))), aes(color=40))
```

Finally, a shift of the moving weight relative to the scale is the only mechanism that describes the observed slow-down of tempo by performers, which in turn can be explained by the user reading the marks below the moving weight.

```
p + scale_color_gradient(breaks=c(0, 12, 16), trans="reverse", name="Scale shift [mm]") +
  stat_function(fun=metr_model_bias, args=list(
    R=R, M.=M., l=l, mu.=mu., A=A, shift=8), aes(color=8)) +
  stat_function(fun=metr_model_bias, args=list(
    R=R, M.=M., l=l, mu.=mu., A=A, shift=12), aes(color=12)) +
  stat_function(fun=metr_model_bias, args=list(
    R=R, M.=M., l=l, mu.=mu., A=A, shift=16), aes(color=16))
```
