# Supplementary figures and images for: Conductors’ tempo choices shed light over Beethoven’s metronome
Source: PLoS One. 2020 Dec 16;15(12):e0243616. doi: 10.1371/journal.pone.0243616 (PMC7743971; doi:10.1371/journal.pone.0243616)

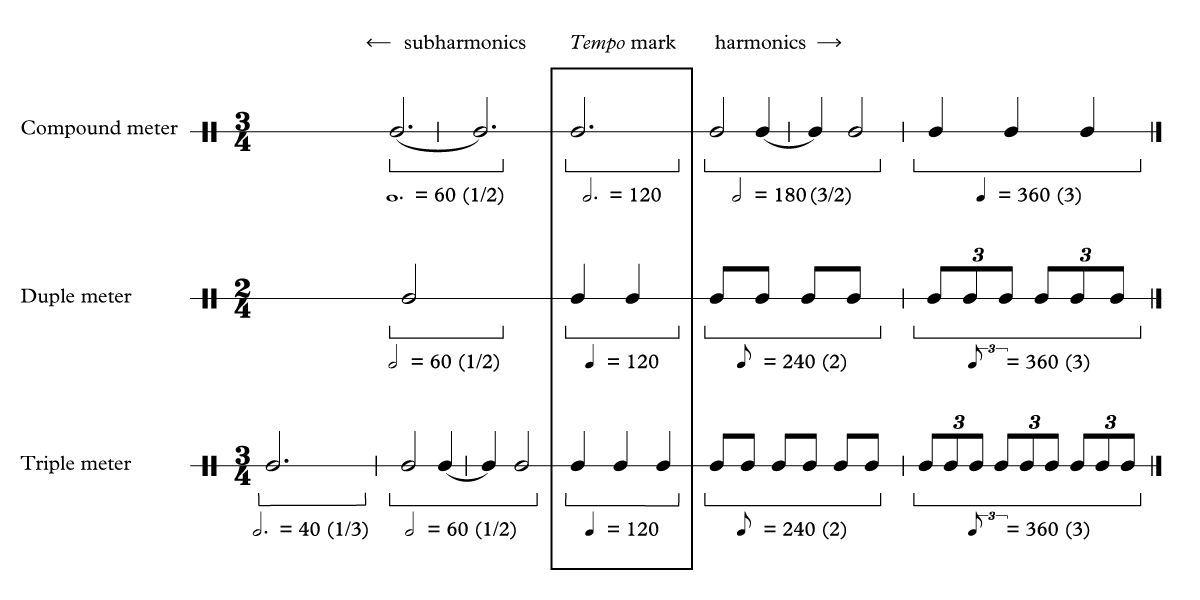

Supplement: S1 File — The bmetr R package contains all supporting data and methods. (GZ) [file pone.0243616.s001.gz › bmetr/inst/extdata/harmonics.png]

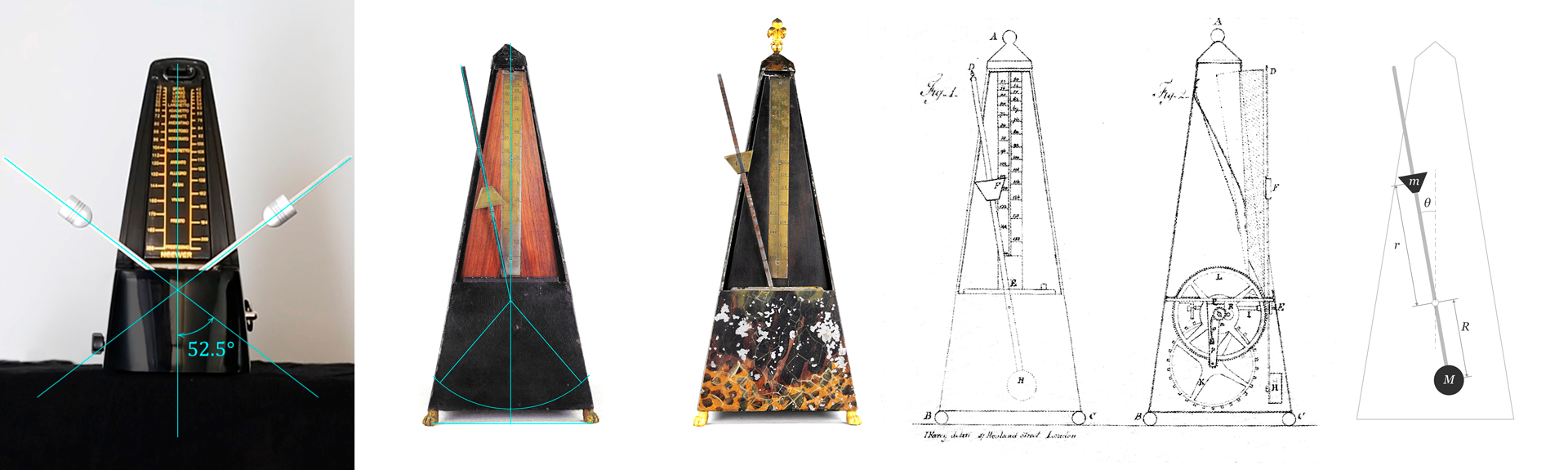

Supplement: S1 File — The bmetr R package contains all supporting data and methods. (GZ) [file pone.0243616.s001.gz › bmetr/inst/extdata/metronomes.jpg]
