# Supplementary material for: Conductors’ tempo choices shed light over Beethoven’s metronome
Source: PLoS One. 2020 Dec 16;15(12):e0243616. doi: 10.1371/journal.pone.0243616 (PMC7743971; doi:10.1371/journal.pone.0243616)
Supplement: Attachment — Submitted filename: response_reviewers.pdf [file pone.0243616.s002.pdf]

Response to reviewers of PONE-D-20-33396

Almudena Martin-Castro, Iñaki Ucar

We are very grateful for a quick yet thorough review, as well as for the kind words about our work. In the following, we provide a response to each comment, explaining how it has been addressed in the new version of the paper. For convenience, we provide a revised version with tracked changes along with the untracked version.

1. *Top of page 3 (line 70), Results. Please spend a few more words about the methods for classifying conductors in the 3 classes*

Thanks for bringing this into our attention. The classification of performances is made by convention: HI are those that use period instruments and follow all the usual HI stylistic criteria (as analysed by Young [7] among others), and usually simply because the conductor explicitly stated the intention to follow such criteria; HI-influenced performers follow the same stylistic guidelines, but may be not so strict in terms of instrumentation; finally, Romantic performances are those previous to the 1980s or, more generally, those that do not adhere to HI performing criteria. These details can be found in the performance reviews included with the recordings (identified in Table 1) as well as Young's PhD thesis [7].

Our intention was to provide a quick note in Results (line 70), and then this description in Methods (line 177), but we forgot to add it. Therefore, we have included this longer explanation to Methods (lines 177-184) in the revised version of the manuscript, because we believe it belongs in this section. However, if bringing such a paragraph forward to Results is considered best for the readership, we would be happy to move it. Note also that we have corrected a broken reference to Table 1 (line 178).

2. *Legend of Figure 3, please define CI (with a 95% CI)*

Added "Confidence Interval" to figure legend as well as line 274.

3. *Fig. 4b. Please indicate where this document is stored (Library, museum etc..)*

Thanks, we forgot to add the reference. The autograph of the 9th Symphony is stored in the Staatsbibliothek in Berlin, and it can be accessed online through a permanent URL. We have added this as reference number 28 in the revised version of the manuscript. We have also added 3 missing URLs in references 25-27.

4. *Please define LRT, line 267, page 9 a significative random effect (LRT =*

Added “Likelihood Ratio Test” to line 275 of the revised version of the manuscript.

5. *Fig 2 and Fig. 4. Can you possibly increase the contrast, rendering the gray more dark?*

We have increased the contrast in both figures, and in Fig 4b we have specified that this is an enhanced version of the image.
